# Responses of the Photosynthetic Electron Transport Reactions Stimulate the Oxidation of the Reaction Center Chlorophyll of Photosystem I, P700, under Drought and High Temperatures in Rice

**DOI:** 10.3390/ijms20092068

**Published:** 2019-04-26

**Authors:** Shinya Wada, Daisuke Takagi, Chikahiro Miyake, Amane Makino, Yuji Suzuki

**Affiliations:** 1 Faculty of Agriculture, Iwate University, 3-18-8 Ueda, Morioka, Iwate 020-8550, Japan; swada@penguin.kobe-u.ac.jp; 2 Graduate School of Agricultural Science, Kobe University, 1-1 Rokkodai, Nada-ku, Kobe 657-8501, Japan; d.takagi@tohoku.ac.jp (D.T.); cmiyake@hawk.kobe-u.ac.jp (C.M.); 3 Graduate School of Agricultural Science, Tohoku University, Aramaki-Aoba 468-1, Aoba-ku, Sendai 980-8572, Japan; amanemakino@tohoku.ac.jp

**Keywords:** drought stress, high temperature, excess light stress, photochemistry of photosystem II and I, rice

## Abstract

It is of interest how photosynthetic electron transport (PET) reactions respond to excess light energy caused by the combination of drought stress and high temperatures. Since such information is scarcely available for photosystem I (PSI), this question was explored in rice (*Oryza sativa* L.) plants subjected to drought stress, using culture solutions that contain poly(ethylene glycol) at different concentrations under two day/night temperature regimes. At 27/22 °C (day/night), drought stress led to the oxidation of the reaction center of the chlorophyll of PSI (P700), and also led to decreases in the quantum efficiencies of photosystem II (PSII) and PSI, and a reduction of the primary quinone electron acceptor of PSI. Such drought stress responses were wholly stimulated at 35/30 °C. These parameters were strongly correlated with each other and were minimally affected by temperature. These results indicate that the drought stress responses of the respective PET reactions are closely associated with each other in the oxidization of P700 and that such responses are stimulated at high temperatures. The underlying mechanisms of these phenomena were discussed. While P700 oxidation is thought to suppress reactive oxygen species (ROS) production, PSI photoinhibition was observed under severe stress conditions, implying that P700 oxidation is not sufficient for the protection of PSI under drought stress.

## 1. Introduction

Drought is one of the most serious abiotic stresses experienced by plants and is often accompanied by high temperatures. How the photosynthetic machinery responds to these stress conditions is of interest, as such information would be important for understanding and improving the stress tolerance of plants. Drought stress can lead to the generation of reactive oxygen species (ROS), which impair photosynthetic machinery. Stomatal closure in response to drought stress prevents water loss via transpiration but yields excess light energy, as decreases in CO_2_ availability within the leaves lead to decreases in energy consumption by the Calvin-Benson cycle [1]. Excess light energy can over-reduce the photosynthetic electron transport (PET) chain and lead to the generation of ROS [2,3]. Photosystem II and I (PSII and PSI, respectively) are known to be the generation sites of ROS [4,5,6]. Excess photons in PSII can generate triplet chlorophyll, with the subsequent formation of highly reactive singlet oxygen. It has been suggested that the over-reduction of the reaction center chlorophyll of PSI (P700) leads to the generation of ROS such as superoxide anions, hydroxyl radicals, and singlet oxygens (see [7], and the literature cited). The combination of drought stress and high temperatures can lead to the over-accumulation of ROS [8]. PSII photoinhibition is repaired efficiently in a short period of time [9,10]. In contrast, PSI photoinhibition, which does not occur frequently, requires a long period of recovery and severely affects photosynthesis and plant growth [11,12]. Therefore, we are interested primarily in the drought responses of the PSI photochemistry under high temperatures. 

PSI is thought to be protected from excess light stress via the oxidation of P700, as indicated by increases in the quantum yield of the donor side limitation of PSI [Y(ND)] (see [13,14]). When P700 was in a more oxidized state under high irradiance, PSI was more tolerant to stress from strong excess light imposed by the repetitive illumination of a saturated pulse-light [13]. The photosynthetic processes, upstream and downstream of PSI, are thought to contribute to P700 oxidation. In the former case, P700 oxidation is promoted by decreasing electron flow to PSI. For example, excess light energy is dissipated as heat at PSII in a light-induced manner, namely, by non-photochemical quenching (NPQ) and a non-light-induced quenching process [5,15,16,17]. It has been suggested that NPQ contributes to the limitation of the downstream electron flow in the oxidation of P700 [18,19,20,21,22,23]. Electron flow is also limited at the cytochrome *b*_6_/*f* complex [23,24]. Luminal acidification is thought to play a regulatory role in NPQ induction and in limiting the electron flow at the cytochrome *b_6_*/*f* complex. It has also been suggested that the over-reduction of the plastoquinone pool suppresses the Q cycle and electron flow at the cytochrome *b*_6_/*f* complex in cyanobacteria, namely, the reduction-induced suppression of electron flow (RISE) [25,26]. The downstream processes of PSI, including photorespiration [27,28,29] and the reduction of O_2_ to H_2_O by flavodiiron proteins [30,31,32,33], are also thought to contribute to P700 oxidation.

When plants underwent drought stress, P700 oxidation was reported to be enhanced concomitantly with decreases in the quantum efficiency of PSII [Y(II)], which in turn were accompanied by increases in NPQ, a reduction of the plastoquinone pool, and decreases in the quantum efficiency of PSI [Y(I)] [1,18,20,21,22,34,35,36]. These results suggest that the PET reactions, upstream of PSI, cooperatively respond to drought stress in order to protect P700 from over-reduction. It is also suggested that electron flow toward PSI is limited because of decreases in the amount of cytochrome *b_6_*/*f* complex [35]. High air temperatures were reported to stimulate the drought stress responses of PSII, triggering decreases in the maximal quantum efficiency of PSII (*F*_v_/*F*_m_) and increases in NPQ [8,37,38,39,40,41]. 

However, it is still not clear whether (or how) PSI responds to the combination of drought and high temperatures, although this constitutes basic, important information on the protective mechanism of PSI. In order to explore this question, we determined the changes in the photochemistry of PSII and PSI and the rate of CO_2_ assimilation (*A*) in the leaves of drought-stressed rice (*Oryza sativa* L.) plants under normal and high air temperature conditions. How responses of the PET reactions under drought stress were regulated was discussed in connection with the relationships between the measured parameters and the amount of cytochrome *f* subunit of the cytochrome *b_6_*/*f* complex. In addition, the responses of the redox-state of P700 were compared with the maximal activity of PSI to assess the contribution of P700 oxidation to the protection of PSI from photoinhibition under drought stress.

## 2. Results

### 2.1. Effects of Drought Stress at Different Temperatures on the Relative Water Contents of Leaves and A

**Figure 1:**
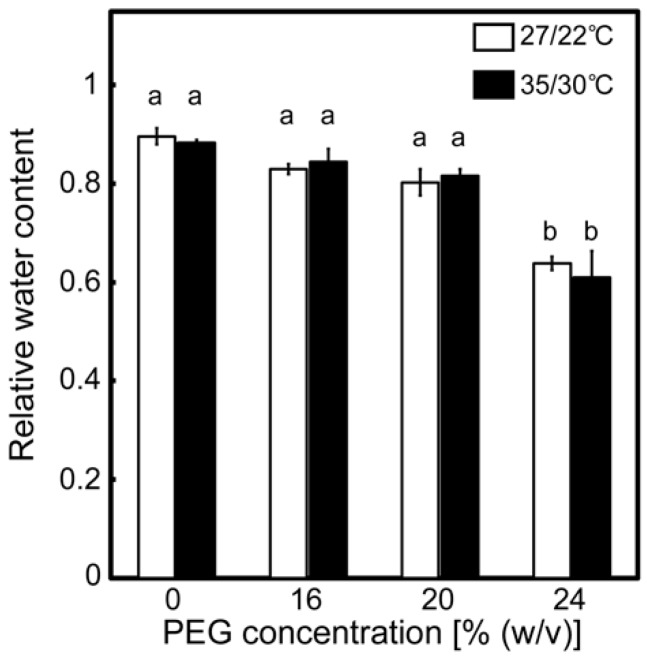
Relative water content of leaves after the drought stress treatment under two different temperature conditions in rice. Approximately 60 days after germination, hydroponically grown plants were drought-stressed using culture solutions containing PEG at 0, 16, 20, and 24% (*w*/*v*) for two days under an irradiance of 450 μmol photon m^−2^ s^−1^ and day/night temperature regimes of 27/22 °C and 35/30 °C. On the day following the stress treatment, the uppermost fully expanded leaves were collected for analysis. White and black bars denote plants stressed under the 27/22 °C and 35/30 °C conditions, respectively. Data are presented as means ± SE (*n* = 3 to 5). Statistical analysis was carried out by ANOVA, followed by Turkey–Kramer’s test. Columns with the same letter were not significantly different (*p* < 0.05).

Drought stress treatments were carried out, following the methods described in Zhou et al. [34], with some slight modifications. Plants were treated with a culture solution containing poly(ethylene glycol) (PEG) at different concentrations for two days. A growth chamber was used to maintain consistent air temperatures and irradiance during the drought stress treatments. The conditions were a day/night temperature of 27/22 °C (normal temperature) or 35/30 °C (high temperature) and an irradiance of 450 μmol photon m^−2^ s^−1^. Drought stress treatments induced the wilting of the leaves on the first day of treatment. Wilting was enhanced as [PEG] in the culture solutions increased at 27/22 °C, and it tended to be further enhanced at 35/30 °C. The extent of this symptom remained mostly unchanged during the treatments. The relative water content of the leaves after stress treatments decreased as [PEG] increased under both temperature conditions (Figure 1). The values recorded at 27/22 °C were comparable with those previously reported [34]. Decreases in the relative water content were not stimulated at 35/30 °C, as had been previously observed in soil culture experiments with rice [42]. These results show that the leaf water status was negatively affected by the drought stress treatments in the present study.

**Figure 2:**
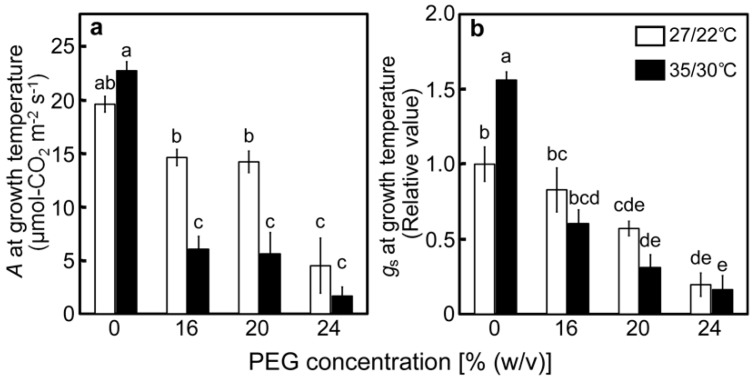
Rate of CO_2_ assimilation (*A*) (**a**) and stomatal conductance (*g*_s_) (**b**) after drought stress treatment under two different temperature conditions in rice. Approximately 60 days after germination, hydroponically grown plants were drought-stressed using culture solutions containing PEG at 0, 16, 20, 24% (*w*/*v*) for two days under an irradiance of 450 μmol photon m^−2^ s^−1^ and day/night air-temperatures of 27/22 °C or 35/30 °C, followed by measurements of *A* and *g*_s_. Measurements were made under conditions of an actinic light intensity of 1250 μmol photon m^−2^ s^−1^, [CO_2_] of 40 Pa, leaf temperature of 27 °C (white bar) or 35 °C (black bar), which corresponded to the daytime air temperature during stress treatment, and relative humidity of 60–70%. Data are presented as means ± SE (*n* = 3 to 5). Statistical analysis was carried out by ANOVA, followed by Turkey-Kramer’s test. Columns with the same letter were not significantly different (*p* < 0.05).

Photosynthetic parameters were measured at the end of the drought stress treatments under the conditions of light saturation and temperatures during drought stress treatments to assess the maximal photosynthetic activity under each stress condition. When gas-exchange parameters were examined, *A* gradually decreased as the [PEG] in the culture solutions increased at 27/22 °C (Figure 2a), whereas the decrease in *A* was accompanied by decreases in stomatal conductance (*g*_s_) (Figure 2b) [1]. At 35/30 °C, *A* and *g*_s_ of the PEG-untreated control plants tended to be higher than those at 27/22 °C (Figure 2a,b). Decreases in *A* and *g*_s_ were stimulated under high-temperature conditions, as previously observed [40,41,43,44]. However, air temperature had a minimal effect on the relative water content (Figure 1). These results mean that the Calvin–Benson cycle was inhibited by the stomatal closure caused by drought stress treatments and that the effects were stronger under high-temperature conditions.

### 2.2. Responses of the PET Reactions

**Figure 3:**
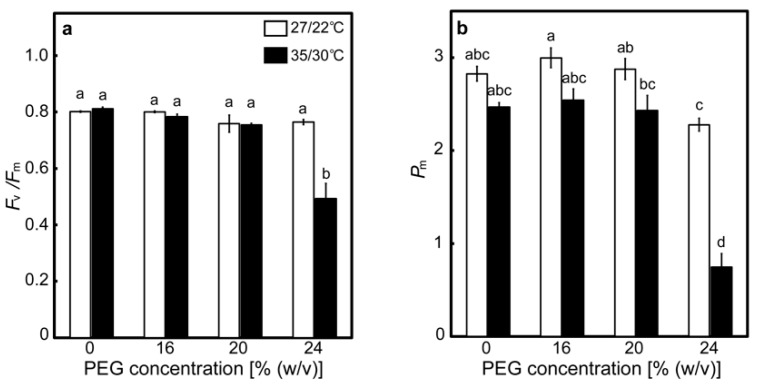
The maximum quantum efficiency of PSII photochemistry (*F*_v_/*F*_m_) (**a**) and the maximal P700 signal (*P*_m_) (**b**) after drought stress treatment under two different temperature conditions in rice. Approximately 60 days after germination, hydroponically grown plants were drought-stressed using culture solutions containing PEG at 0, 16, 20, 24% (*w*/*v*) for two days under an irradiance of 450 μmol photon m^−2^ s^−1^ and day/night air-temperatures of 27/22 °C or 35/30 °C. Leaves were dark-adapted for more than 30 min, followed by the measurements of *F*_v_/*F*_m_ and *P*_m_ under conditions of [CO_2_] of 40 Pa, leaf temperature of 27 °C (white bar) or 35 °C (black bar), which corresponded to the daytime air temperature during the stress treatment, and relative humidity of 60–70%, without the illumination of actinic light. Data are presented as means ± SE (*n* = 3 to 5). Statistical analysis was carried out by ANOVA, followed by Turkey–Kramer’s test. Columns with the same letter were not significantly different (*p* < 0.05).

The effects of drought stress at different temperatures on the maximum quantum efficiency of PSII photochemistry (*F*_v_/*F*_m_) and the maximal P700 signal (*P*_m_) were determined as indices for the photoinhibition of PSII and PSI, respectively (Figure 3). The values of *F*_v_/*F*_m_ were around 0.8 in the control plants and the 16% PEG-treated plants at 27/22 °C. There was no statistically significant difference in the values of *F*_v_/*F*_m_ between the control plants and the PEG-treated plants, although the values of *F*_v_/*F*_m_ tended to marginally decrease in the 20% and 24% PEG-treated plants (Figure 3a). The values of *F*_v_/*F*_m_ were also around 0.8 in the control plants at 35/30 °C, and they tended to marginally decrease in the 16% and 20% PEG-treated plants and drastically decreased to 0.490 in the 24% PEG-treated plants. These results show that considerable PSII photoinhibition occurred only when plants were subjected to severe drought stress at high temperatures. The values of *P*_m_ were around 2.9 in the control plants and the 16% and 20% PEG-treated plants at 27/22 °C but significantly decreased to 2.28 in the 24% PEG-treated plants (Figure 3b). Decreases in *P*_m_ were reported to be greater than those in *F*_v_/*F*_m_ when some tropical evergreen tree species were exposed to a prolonged drought [36], meaning that PSI is more susceptible to drought stress. Similar trends were observed in rice. The values of *P*_m_ were around 2.5 in the control plants and the 16% and 20% PEG-treated plants at 35/30 °C but were drastically decreased to 0.75 in the 24% PEG-treated plants, showing that drought-induced PSI photoinhibition was enhanced at high temperatures.

**Figure 4:**
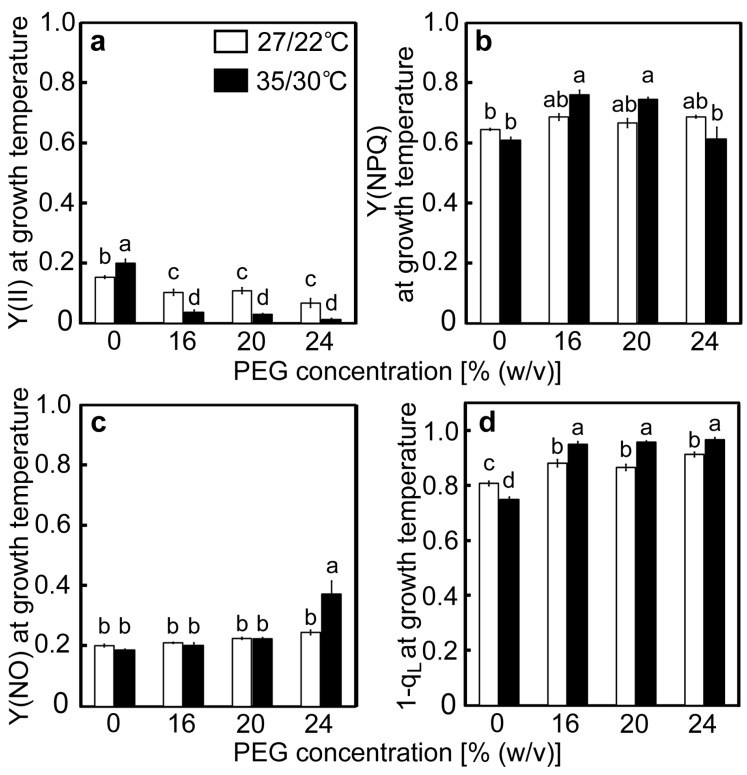
Chlorophyll fluorescence parameters after drought stress treatment under two different temperature conditions in rice. Approximately 60 days after germination, hydroponically grown plants were drought-stressed using culture solutions containing PEG at 0, 16, 20, 24% (*w*/*v*) for two days under an irradiance of 450 μmol photon m^−2^ s^−1^ and day/night air temperatures of 27/22 °C or 35/30 °C. After the measurements of *F*_v_/*F*_m_ and *P*_m_, chlorophyll fluorescence parameters were measured. Panels (**a**), (**b**), (**c**), and (**d**) show the results for Y(II), Y(NPQ), Y(NO), and 1−q_L_, respectively. Measurements were made under conditions of an actinic light intensity of 1250 μmol photon m^−2^ s^−1^, [CO_2_] of 40 Pa, leaf temperature of 27 °C (white bar) or 35 °C (black bar), which corresponded to the daytime air temperature during stress treatment, and relative humidity of 60–70%. Data are presented as means ± SE (*n* = 3 to 5). Statistical analysis was carried out by ANOVA, followed by Turkey–Kramer’s test. Columns with the same letter were not significantly different (*p* < 0.05).

Changes in the photochemistry of PSII were examined in response to drought stress at different temperatures. The value of Y(II) in the control plants at 27/22 °C was 0.153 and tended to decrease as [PEG] in the culture solutions increased (Figure 4a). The quantum yield values of NPQ [Y(NPQ)] and the non-regulated and non-photochemical energy dissipation [Y(NO)] in the control plants were 0.646 and 0.201, respectively (Figure 4b,c). As decreases in Y(II), caused by the PEG treatments, were accompanied by increases in both Y(NPQ) and Y(NO), changes in Y(NPQ) and Y(NO) were marginal. The value of Y(II) in the control plants at 35/30 °C was 0.203, which is higher than that observed at 27/22 °C (Figure 4a). The values of Y(II) greatly decreased in the PEG-treated plants at 35/30 °C and were lower than those in the PEG-treated plants at 27/22 °C. The values of Y(NPQ) and Y(NO) in the control plants were comparable to those at 27/22 °C (Figure 4b,c). Decreases in Y(II) were accompanied by slight increases in Y(NPQ) in the 16% and 20% PEG-treated plants. In contrast, in the 24% PEG-treated plants, where decreases in *F*_v_/*F*_m_ were observed (Figure 3a), Y(NO) showed a two-fold increase, whereas Y(NPQ) remained at the control level. The redox-state of the primary quinone electron acceptor of PSII (Q_A_) was determined by the value of 1−q_L_ (Figure 4d), which stands for the fraction of PS II centers in closed states, according to the definition of Kramer et al. [16]. The 1−q_L_ is also thought to reflect the redox-state of the plastoquinone pool [45]. The values of 1−q_L_ in the control plants were 0.807 and 0.751 at 27/22 °C and 35/30 °C, respectively, and tended to increase as [PEG] in the culture solutions increased. The values of 1−q_L_ were greater at 35/30 °C under all [PEG] conditions.

**Figure 5:**
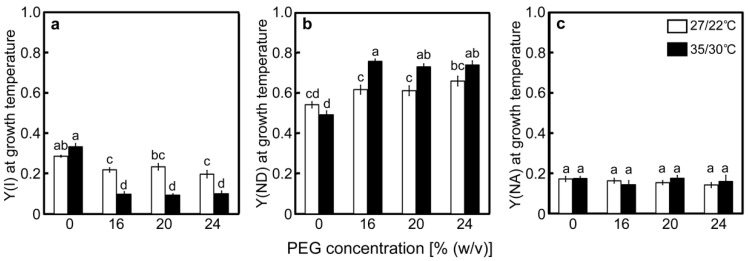
Redox state of P700 after drought stress treatment under two different temperature conditions in rice. Approximately 60 days after germination, hydroponically grown plants were drought-stressed using culture solutions containing PEG at 0, 16, 20, 24% (*w*/*v*) for two days under an irradiance of 450 μmol photon m^−2^ s^−1^ and day/night air temperatures of 27/22 °C or 35/30 °C. P700 absorption was measured simultaneously with chlorophyll fluorescence. Panels (**a**), (**b**), and (**c**) show the results for Y(I), Y(ND), and Y(NA), respectively. Measurements were made under conditions of an actinic light intensity of 1250 μmol photon m^−2^ s^−1^, [CO_2_] of 40 Pa, leaf temperature of 27 °C (white bar) or 35 °C (black bar), which corresponded to the daytime air temperature during stress treatment, and relative humidity of 60–70%. Data are presented as means ± SE (*n* = 3 to 5). Statistical analysis was carried out by ANOVA, followed by Turkey–Kramer’s test. Columns with the same letter were not significantly different (*p* < 0.05).

Changes in the redox state of P700 were examined concurrently with the photochemistry changes of PSII. The value of Y(I) in the control plants at 27/22 °C was 0.286 and tended to decrease as [PEG] in the culture solutions increased (Figure 5a). The values of Y(ND) and the quantum yield of the acceptor side limitation of PSI [Y(NA)], which stand for the rate of P700 excitation, were 0.543 and 0.172, respectively, in the control plants (Figure 5b,c). Y(ND) increased as [PEG] in the culture solutions increased, whereas Y(NA) was unaffected by the PEG treatments. At 35/30 °C, the value of Y(I) in the control plants was 0.325, which was higher than that observed at 27/22 °C (Figure 5a). The values greatly decreased in the PEG-treated plants and were lower than those in the PEG-treated plants at 27/22 °C. The values of Y(ND) and Y(NA) in the control plants were 0.444 and 0.193, respectively (Figure 5b,c). Decreases in Y(I), caused by the PEG treatments, were accompanied by increases in Y(ND), whereas Y(NA) was unaffected. The decreases observed in Y(I), caused by the PEG treatments, primarily reflected the increases in Y(ND) at both temperatures.

### 2.3. Relationships between the Photosynthetic Parameters

**Figure 6:**
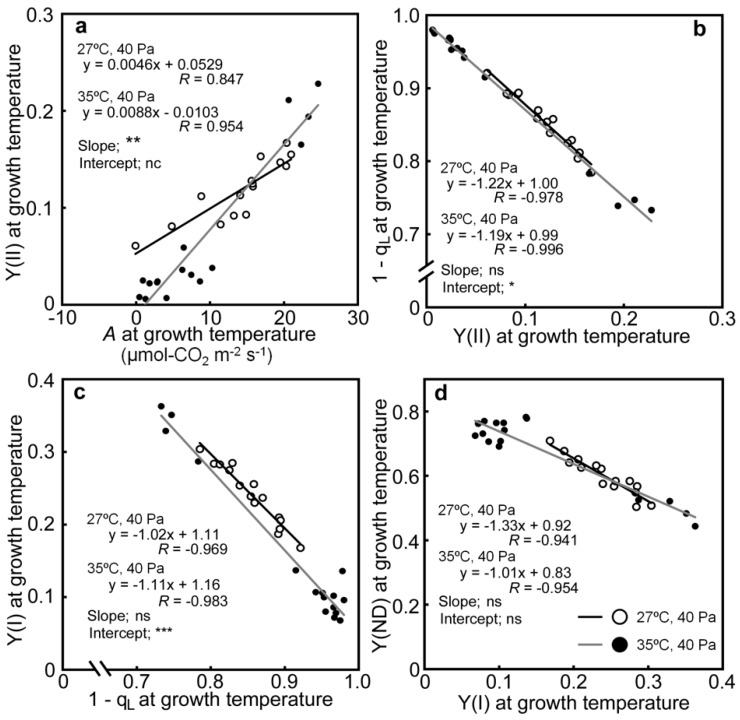
Relationship between Y(II) and *A* (**a**), 1−q_L_ and Y(I) and Y(II) (**b**), Y(I) and 1−q_L_ (**c**), and Y(ND) and Y(I) (**d**). The data are taken from Figure 2, Figure 4, and Figure 5. Regression lines were made from a dataset, obtained at a given air temperature. White and black circles correspond to data obtained from drought-stressed plants at 27/22 °C and 35/30 °C, respectively. The results of statistical treatments between regression lines by ANCOVA were presented in each panel. The slopes of regression lines were tested first. When there were no significant differences, the intercepts of the regression lines were tested. *, **, and *** denote statistically significant differences at *p* < 0.05, 0.01, and 0.001, respectively. The ns and nc notations denote not significant and not calculated, respectively.

The relationships between the photosynthetic parameters were investigated (Table S1). Strong correlations were observed between *A*, Y(II), 1−q_L_, Y(I), and Y(ND). Those correlations are shown in Figure 6. Comparisons were made between Y(II) and *A*, to examine the effects of decreases in *A* on linear electron flow, and between 1−q_L_ and Y(II), Y(I) and 1−q_L_, and Y(ND) and Y(I), to examine the mutual relationships between the successive PET reactions. Y(II) was positively correlated with *A* under both air temperature conditions (Figure 6a), although the relationship was affected slightly by temperature. Y(II) changed proportionally with *A* at 35/30 °C, as the regression line passed near the origin, whereas the changes in Y(II) were smaller at 27/22 °C. Strong negative correlations were observed between 1−q_L_ and Y(II), Y(I) and 1−q_L_, and Y(ND) and Y(I) (Figure 6b–d). While there were statistically significant differences in the relationships between 1−q_L_ and Y(II), and Y(I) and 1−q_L_, depending on the air temperatures, the differences in the slopes and intercepts were small (less than 10%).

### 2.4. Effects of Drought Stress on the Amount of Cytochrome f

**Figure 7:**
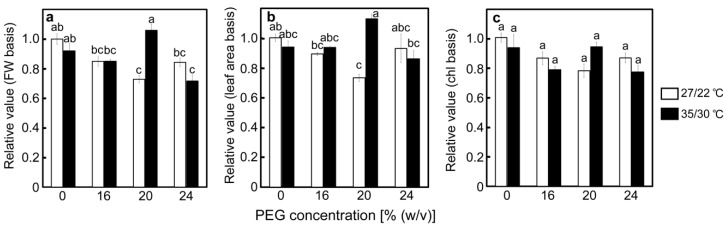
The relative amount of cytochrome *f* after drought stress treatment on a leaf fresh weight basis (**a**), leaf area basis (**b**), and unit chlorophyll content basis (**c**) under two different temperature conditions in rice. Approximately 60 days after germination, hydroponically grown plants were drought-stressed using culture solutions containing PEG at 0, 16, 20, 24% (*w*/*v*) for two days under an irradiance of 450 μmol photon m^−2^ s^−1^ and day/night air temperatures of 27/22 °C or 35/30 °C, followed by western blotting using polyclonal-monospecific antibodies against cytochrome *f*. Data of the PEG-untreated control plants were defined as 1. Data are presented as means ± SE (*n* = 3). Statistical analysis was carried out by ANOVA, followed by Turkey–Kramer’s test. Columns with the same letter were not significantly different (*p* < 0.05).

As it was suggested that decreases in the cytochrome *b_6_*/*f* content limit downstream electron flow and prevent the over-reduction of P700 under drought stress [35], one of its subunits (cytochrome *f*) was determined. The results of the detection of cytochrome *f* by Western blotting, followed by SDS-PAGE, are shown in Figure S1. Relative amounts of cytochrome *f* were shown on a leaf fresh weight basis, a leaf area basis, and a chlorophyll content basis (Figure 7a–c). The amount of cytochrome *f* was relatively stable under stress conditions, with a maximum decrease of approximately 30% observed in the 20% PEG-treated plants at 27/22 °C. Decreases in the amount of cytochrome *f* were not stimulated at 35/30 °C.

## 3. Discussion

### 3.1. Drought Stress Responses of the PET Reaction that Leads to P700 Oxidization Are Stimulated under High Temperatures

We tried to characterize drought stress responses of the PET reactions, focusing especially on the redox state of PSI at high temperatures in rice. At normal temperatures, it is the drought stress responses of the PET reactions are to limit the electron flow to PSI [1,18,20,21,22,34,35,36]. Our results at 27/22 °C were consistent with these previous reports. Decreases in *A* and *g*_s_ under drought stress led to the limitation of the linear electron flow, as indicated by the decreases in Y(II) (Figure 2 and Figure 4a). Subsequently, the reduction of Q_A_, decrease in the electron flow at PSI, and oxidation of P700 occurred, as indicated by the increases in 1−q_L_, decreases in Y(I), and increases in Y(ND), respectively (Figure 4 and Figure 5). In the present study, it was further found that these drought stress responses were wholly stimulated under the high-temperature conditions of 35/30 °C (Figure 4 and Figure 5). Interestingly, these PET reaction parameters were strongly correlated with each other and were minimally affected by temperature during stress treatments (Table S1; Figure 5). These results indicated that the drought stress responses of the respective PET reactions were closely associated with each other in the oxidation of P700 and that such responses are stimulated at high temperatures.

The photoinhibition of PSI or PSII and PSI was observed in the 24% PEG-treated plants at 27/22 °C or 35/30 °C (Figure 3). Whether this photoinhibition affected the drought stress response of the PET reactions is an issue. The strong correlations between the parameters of the PET reactions were observed, even in the presence of photoinhibition (Table S1; Figure 6), implying that the effects of the photoinhibition were small in the present experimental condition. The following previous observation might support this assumption. Decreases in *F*_v_/*F*_m_ led to decreases in *A*, but such effects became small under low [CO_2_] conditions in *Chenopodium album* [46], implying that linear electron flow was not greatly affected by *F*_v_/*F*_m_ when *A* was suppressed. Parameters, such as Y(II) and *A*, tended to be greatly affected by drought stress conditions, rather than by *F*_v_/*F*_m_, in tomato and citrus [38,39,40]. On the other hand, it has been reported that the specific photoinhibition of PSI affected other photosynthetic processes, including decreases in Y(II), the reduction of Q_A_, and decreases in *A* in wheat and Arabidopsis, when measured under normal or elevated [CO_2_] conditions [47,48]. However, it is still unknown how these parameters respond when *A* is severely suppressed. At least, Y(II) and *A*, measured under high irradiance, also seemed to be affected by drought stress conditions, rather than by *P*_m_, in some tropical tree species [36].

### 3.2. Possible Mechanisms of the Drought Stress Responses of the PET Reactions at High Temperatures

It is of interest how the drought stress response of the PET reactions was regulated at high temperatures. Luminal acidification would be one candidate for the regulatory factors, as it induces NPQ at PSII [5,15,20] and slows down the oxidation of plastoquinol by the cytochrome *b_6_*/*f* complex [23,24,49]. Such regulations are thought to occur under drought stress at non-stressful temperatures [22,35]. A decrease in q_L_ could reflect luminal acidification as these phenomena were simultaneously observed when the amounts of the chloroplastic ATP synthase were decreased in transgenic and transplastomic tobacco plants [23]. In the present study, the values of 1−q_L_ increased and were strongly correlated with other parameters of the PET reactions, irrespective of the temperature (Table S1; Figure 4d and Figure 6). These results imply the regulation of the drought stress responses of the PET reactions by luminal acidification at high temperatures in rice. Increases in 1−q_L_ are also thought to reflect a reduction of the plastoquinone pool [45], which was suggested to play a regulatory role in the PET reactions when photosynthesis was inhibited. In cyanobacteria, cultured under CO_2_-depleted conditions, an over-reduction of the plastoquinone pool was shown to inhibit the Q cycle at the cytochrome *b*_6_/*f* complex and limit the downstream electron flow, leading to P700 oxidation [25,26]. It might be reasonable to assume that the same regulatory mechanism operated under drought stress at high temperatures in rice, although it remains unclear whether this mechanism is operative in vascular plants. In addition, decreases in the amount of cytochrome *b_6_*/*f* complex have been suggested to limit the electron flow to PSI in response to drought stress at non-stressful temperatures. The amount of cytochrome *b_6_*/*f* complex, as well as the linear electron flow at PSII in drought-stressed wild watermelon plants, decreased to approximately 50% of the levels observed in non-stressed plants [35]. Such a substantial decrease in the cytochrome *f* content was not observed in the present study (Figure S1 and Figure 7), and it is unlikely that such regulation occurred in rice, irrespective of the temperature. The difference between these studies may be attributed to different strategies for protecting PSI under drought stress conditions in different plant species.

It has also been suggested that P700 oxidation under drought stress is related to operation of the cyclic electron flow around PSI (CEF-PSI), leading to the formation of luminal acidification, induction of NPQ, and suppression of ROS production in PSII [18,50]. The rationale for the operation of CEF-PSI was the observation that Y(I) was higher than Y(II) [18,20,21,22]. Similar results were also observed in the present study (Figure 4a and Figure 5a). To assess whether CEF-PSI is operative under drought stress in more detail, the simultaneous monitoring of the redox state of P700, plastocyanin, and ferredoxin, as well as chlorophyll fluorescence [51,52], would be useful. Recently, Takagi and Miyake [53] used this system to determine the ratios of the linear electron flow rates to the ferredoxin oxidation rates as an index of the activity of ferredoxin-dependent CEF-PSI in Arabidopsis. It was found that the activity of ferredoxin-dependent CEF-PSI was minor under normal conditions. How this situation changes under drought stress needs to be studied.

While the changes in Y(II) were positively correlated with those in *A* under drought stress, temperature affected this relationship. The decreases in Y(II) were relatively smaller than those in *A* at 27/22 °C (Figure 2a, Figure 4a and Figure 6a), agreeing with previous reports on drought stress at normal temperatures [1,18,21,22]. Such phenomena are related to the operation of photorespiration. While *A* decreased because of drought stress, the rate of energy consumption by photorespiration was shown to increase [54,55,56,57,58], leading to the maintenance of Y(II), unlike *A*. On the other hand, the regression line between Y(II) and *A* passed near the origin at 35/30 °C (Figure 6a), indicating that Y(II) was primarily dependent on *A*. One explanation might be the inhibition of photorespiration. Considerable decreases in Y(II) were also observed when CO_2_ assimilation and photorespiration were inhibited under the combination of low [CO_2_] and low [O_2_] conditions [28]. It is unlikely that PSI and PSII photoinhibition greatly affected the relationship between Y(II) and *A*, as mentioned before. It was reported that some photorespiratory enzymes were unaffected even under severe drought stress, whereas those of the Calvin–Benson cycle, such as NADP-dependent glyceraldehyde-3-phosphate dehydrogenase and fructose-1,6-bisphosphatase, decreased [54]. In the present study, it is possible that some unknown inhibition of the photorespiratory pathway occurred under the combination of drought stress and high temperatures. On the other hand, it is also possible that photorespiration operated similarly under drought stress at high temperatures. It has previously been observed that Y(NA) increased under CO_2_-limited conditions when the activity of CO_2_ assimilation/photorespiration was decreased by the antisense suppression of Rubisco content in rice [29]. Such changes in Y(NA) were not observed in the present study (Figure 5c). Further validation through biochemical assays will be necessary to assess this hypothesis concerning the inhibition of photorespiration.

### 3.3. P700 Oxidation Is not Sufficient for the Protection of PSI from Photoinhibition under Drought Stress

P700 oxidation is thought to contribute to the prevention of PSI photoinhibition via the suppression of ROS production at PSI [13,14]. While Y(ND) tended to increase as stress became strong (Figure 5b), PSI photoinhibition was observed in the 24% PEG-treated plants at both temperatures (Figure 3b), meaning that P700 oxidation is not sufficient to fully prevent PSI photoinhibition under drought stress at high and normal temperatures. Previous reports have implied that ROS generation and oxidative damage is unavoidable in leaves. Even when P700 oxidation was stimulated in the presence of strong actinic light illumination, excess light stress, imposed by repetitive illumination of saturated pulse light, decreased *P*_m_ to some extent in some plant species, including rice [13,14,29]. While ROS generation can be greatly suppressed by P700 oxidation, ROS, unavoidably generated, might be harmful under drought stress. It has also been suggested that mechanisms, other than P700 oxidation, are involved in protection against ROS. For example, the tolerance of PSI to the repetitive illumination of saturated pulse light differed among angiosperms when it was imposed in the absence of actinic light illumination and P700 oxidation was not induced [14]. In addition, severe drought stress increased the rate of mitochondrial respiration to compensate for decreased ATP production in chloroplasts, leading to enhanced ROS production in mitochondria [59]. ROS accumulation in mitochondria can lead to programmed cell death [60]. Such mechanisms might have been affected by drought stress, leading to damage to the PSI. Further investigation is necessary to unveil the mechanisms of PSI photoinhibition under drought stress and its temperature dependence.

## 4. Conclusions

Drought stress decreased *A* and *g*_s_, triggering the oxidation of P700 via the responses of the PET reactions, such as decreases in Y(II), the reduction of Q_A_, and decreases in Y(I), in rice. Such responses were wholly stimulated under drought stress at high temperatures. Strong correlations between the PET reactions parameters indicate that drought stress responses of the PET reactions are closely associated with each other, irrespective of temperature, leading to the stimulation of the whole drought stress response at high temperatures. Luminal acidification and/or the reduction of the plastoquinone pool may be potential regulatory factors of the drought stress response of the PET reactions at high temperatures. It is also implied that the contribution of photorespiration to the consumption of excess light energy was decreased under drought stress at high temperatures due to its inhibition. P700 oxidation is expected to protect PSI from photoinhibition. However, although P700 oxidation was stimulated, PSI photoinhibition was observed under severe drought stress, meaning that P700 oxidation is not sufficient for the protection of PSI from photoinhibition under drought stress. The detailed mechanism of the drought stress responses at high temperatures remain unknown and require further study. Whether similar drought stress responses are observed at high temperatures in other plant species is also of interest and needs to be studied.

## 5. Materials and Methods

### 5.1. Plant Materials and Growth Conditions

Rice (*Oryza sativa* L. ‘Notohikari’) plants were hydroponically grown in an air-conditioned greenhouse (S-153A, Koito Manufacturing Co. Ltd., Tokyo, Japan) under natural sunlight conditions. Light was supplemented from 0500 to 1900 by two LED lamp units (1000 W Hydroponic Growing Lighting LED Plant Light, Chonquing Hanfan Technology Co. Ltd., Chongqing, China) outputting 200 μmol photon m^−2^ s^−1^ at plant height. Day/night temperatures were 27/22 °C. After germination and culturing in tap water for 20 days, each seedling was transplanted to a 1.1 L plastic pot containing a nutrient solution, as previously described in [61]. The nutrient solution was renewed once a week. The strength of the nutrient solution was increased, depending on the plant growth. The uppermost fully expanded leaves were used for the experiments, from approximately 60 days after germination, when the 11th leaf on the main culm was almost fully expanded.

### 5.2. Drought Stress Treatment

Drought stress treatments were carried out, following the methods of Zhou et al. [34]. Approximately 60 days after germination, when the 11th leaf of the main culm was fully expanded, the plants were transferred to environmentally controlled growth chambers, operated with two different air-temperature conditions and fed nutrient solutions containing PEG at different concentrations for two days to induce drought stress. The growth chambers (NC-411HC, NK System, Osaka, Japan) were operated with an irradiance of 450 μmol photon m^−2^ s^−1^, a 14 h photoperiod, and a day/night air-temperature of 27/22 °C and 35/30 °C for the normal and high-temperature conditions, respectively. PEG, with an average molecular weight of 6000 (PEG, Sigma-Aldrich, St. Louis, MO, USA), was used in all treatments. The concentrations of PEG used in the culture solutions were 16, 20, and 24% (*w*/*v*). A nutrient solution without PEG was used as the control treatment. The day after the drought stress treatment, P700 absorbance and chlorophyll fluorescence parameters were measured, as described below.

### 5.3. Measurements of the Relative Water Content of Leaves

The relative water content of the leaves was determined after the stress treatment, following the methods of Zhou et al. [34]. Leaves were collected, immediately weighed (FW_1_), immersed overnight in deionized water at 4 °C, and weighed again (FW_2_). The dry weight (DW) of the leaves was measured, after being dried at 70 °C for at least 3 days. The relative water content was calculated as (FW_1_ − DW)/(FW_2_ − DW).

### 5.4. Measurements of Photosynthesis

Chlorophyll fluorescence, P700 absorbance, and *A* were simultaneously measured using a GFS-3000 and a DUAL-PAM-100 m conjunction system (Heinz Walz GmbH, Effeltrich, Germany). Plants were kept in the dark for at least 30 min prior to the measurements. Measurements were initiated at a [CO_2_] of 40 Pa. The leaf temperature was either 27 °C or 35 °C, corresponding to the air temperature during stress treatment. The relative air humidity was 60 to 70%. The minimal fluorescence in the dark-adapted state (*F*_o_) was recorded after the illumination of a weak measuring light (620 nm) at a photon flux density of 0.05–0.1 μmol photon m^−2^ s^−1^. A saturating pulse light (300 ms, 10,000 μmol photon m^−2^ s^−1^) was applied to determine the maximal fluorescence in the dark-adapted state (*F*_m_). Leaves were equilibrated under actinic light illumination (1250 μmol photon m^−2^ s^−1^). The maximal and minimal fluorescence in the light-adapted state (*F*_m_’ and *F*_o_’) and steady-state chlorophyll fluorescence (*F*_s_) were recorded after 20 to 30 min of exposure to the actinic light illumination. Prior to measuring *F*_m_’ and *F*_o_’, a saturating pulse light and far-red light (720 nm) were applied, respectively. Chlorophyll fluorescence parameters were calculated, following the methods of Kramer et al. [16] and Baker [62]. The maximal quantum yield of PSII and NPQ were calculated as *F*_v_/*F*_m_ and (*F*_m_ − *F*_m_’)/*F*_m_’, respectively. The index for the reduction of the primary plastoquinone electron acceptor (Q_A_) (1−q_L_) was calculated as 1 − (*F*_m_’ − *F_s_*)/(*F*_m_’ − *F*_o_’) x (*F*_o_’/*F_s_*). Y(II), Y(NO), and Y(NPQ) were calculated as (*F*_m_’ − *F_s_*)/*F*_m_’, 1/[NPQ + 1 + q_L_(*F*_m_/*F*_o_ − 1)], and 1 − Y(II) − 1/[NPQ + 1 + q_L_(*F*_m_/*F*_o_ − 1)], respectively. Three complementary quantum yields were defined: Y(II) + Y(NO) + Y(NPQ) = 1. The redox change of P700 was assessed, according to Klughammer and Schreiber [63], as described in [64], simultaneously with the chlorophyll fluorescence measurements. The maximal P700 signal (*P*_m_) was determined by the application of a saturated pulse light in the presence of far-red light (720 nm), while that of the oxidized P700 during actinic light illumination (*P*_m_’) was determined by a saturated pulse-light application. The P700 signal during actinic light illumination (*P*) was recorded just prior to the saturated pulse light application. Y(I), Y(NA), and Y(ND) were calculated as (*P*_m_’ – *P*)/*P*_m_, (*P*_m_ – *P*_m_’)/*P*_m_, and *P*/*P*_m_, respectively. Three complementary quantum yields were defined: Y(I) + Y(NA) + Y(ND) = 1. *A* and *g*_s_ were also simultaneously recorded. The values of *g*_s_ were presented as relative values, where the values of the PEG-untreated control plants at 27/22 °C were defined as 1.

### 5.5. Determination of Cytochrome f Contents

After the drought stress treatment, leaves equivalent to those used for the photosynthetic measurements were collected, frozen in LN_2_, and stored at −80 °C, after the leaf area and fresh weight were measured. Samples were homogenized with sodium-phosphate buffer, and the amount of chlorophyll was measured. Treatments with SDS were performed as described in [65] but used the leaf homogenate for SDS treatment, instead of the supernatant of the leaf homogenate. Western blotting was conducted as described in [66], with modifications. An aliquot of each SDS-treated sample, corresponding to a leaf fresh weight of 0.09 mg, were loaded onto a gel. Different volumes of SDS-treated samples, prepared from the PEG-untreated control plants at 27/22 °C, corresponding to leaf fresh weights of 0.03, 0.06, 0.12, and 0.18 mg, were loaded onto a gel to generate a calibration curve. Polyclonal-monospecific antibodies against cytochrome *f* [67] were used for immunological detection. The signal intensity was normalized on a leaf area basis or a chlorophyll content basis.

### 5.6. Statistical Treatment

Three to five biological replicates were used. The data shown in Figure 1, Figure 2, Figure 3 and Figure 4 were statistically analyzed using the Tukey–Kramer’s HSD test with JMP11 (SAS Institute Japan, Tokyo, Japan). For the data shown in Figure 5, the Pearson correlation coefficients of the measured parameters and their *P*-values were calculated using Microsoft Excel. Statistically significant differences between the regression lines in Figure 5 were tested using an analysis of covariance (ANCOVA). The slopes of the regression lines were tested first, and the intercepts were tested if no significant differences were determined. Analyses were carried out using R [68].

## Supplementary Materials

Supplementary materials can be found at https://www.mdpi.com/1422-0067/20/9/2068/s1.

## Abbreviations

A	the rate of CO_2_ assimilation
CEF-PSI	cyclic electron flow around photosystem I
gH^+^	the conductance of H^+^ across the thylakoid membranes
*g*_s_	stomatal conductance
NPQ	non-photochemical quenching
P700	the reaction center chlorophyll of photosystem I
PEG	poly (ethylene glycol)
PET	photosynthetic electron transport
PSII	photosystem II
PSI	photosystem I
Q_A_	the primary quinone electron acceptor of photosystem II
RISE	reduction-induced suppression of electron flow
ROS	reactive oxygen species
Y(II)	the quantum efficiency of photosystem II
Y(NO)	the quantum yield of non-regulated and non-photochemical energy dissipation at photosystem II
Y(NPQ)	the quantum yield of non-photochemical quenching at photosystem II
Y(I)	the quantum efficiency of photosystem I
Y(NA)	the quantum yield of the acceptor side limitation of photosystem I
Y(ND)	the quantum yield of the donor side limitation of photosystem I
