# Supplementary material for: Responses of the Photosynthetic Electron Transport Reactions Stimulate the Oxidation of the Reaction Center Chlorophyll of Photosystem I, P700, under Drought and High Temperatures in Rice
Source: Int J Mol Sci. 2019 Apr 26;20(9):2068. doi: 10.3390/ijms20092068 (PMC6539619; doi:10.3390/ijms20092068)
Supplement: Supplementary file 1 [file ijms-20-02068-s001.pdf]

Table S1. Pearson correlation coefficients among the parameters measured in the present study. Data obtained under different conditions of air temperature were analyzed together. ** and *** denote statistically significance at P < 0.01 and P < 0.001, respectively.

	g_s	Y(II)	Y(NPQ)	Y(NO)	1-q_L	Y(I)	Y(ND)	Y(NA)
A	0.904***	0.925***	-0.384*	-0.500**	-0.916***	0.900***	-0.864***	0.113
g_s		0.837***	-0.359*	-0.444*	-0.838***	0.793***	-0.741***	0.037
Y(II)			-0.441*	-0.517**	-0.994***	0.982***	-0.955***	0.164
Y(NPQ)				-0.540**	0.457**	-0.542**	0.444*	0.164
Y(NO)					0.496**	-0.404*	0.472**	-0.309
1-q_L						-0.975***	0.950***	-0.169
Y(I)							-0.946***	0.083
Y(ND)								-0.403*

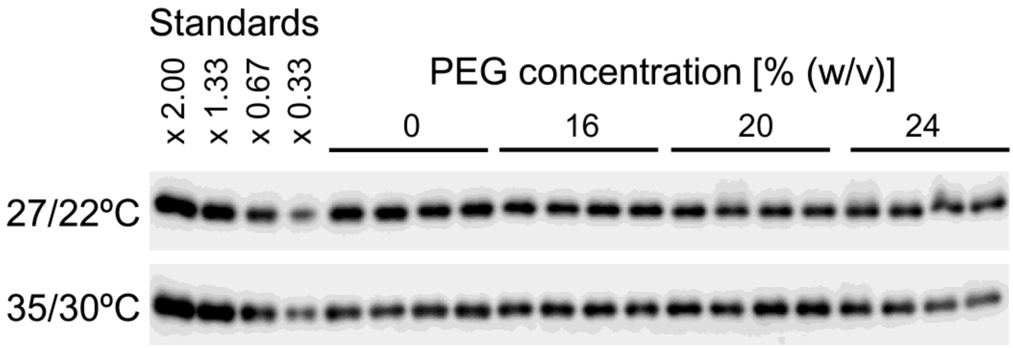

Figure S1. Immunological detection of cytochrome *f* after drought stress treatment under two different temperature conditions in rice. Approximately 60 days after germination, hydroponically grown plants were drought-stressed using culture solutions containing PEG at 0, 16, 20, 24% (w/v) for two days under an irradiance of 450 μmol photon m^{-2} s^{-1} and a day/night air-temperatures of 27/22°C or 35/30°C, followed by Western blotting using polyclonal-monospecific antibodies against cytochrome *f*. An aliquot of each SDS-treated sample, corresponding to a leaf fresh weight of 0.09 mg, were loaded onto a gel. To generate a calibration curve (designated as Standards in the figure), 0.33- to 2.00-fold volume of the sample prepared from the PEG-untreated plants at 27/22°C were loaded onto a gel.
